# Multi-Channel Capacitive Sensor Arrays

**DOI:** 10.3390/s16020150

**Published:** 2016-01-25

**Authors:** Bingnan Wang, Jiang Long, Koon Hoo Teo

**Affiliations:** 1 Mitsubishi Electric Research Laboratories, 201 Broadway, Cambridge, MA 02139, USA; jilong@ucsd.edu (J.L.); teo@merl.com (K.H.T.); 2 University of California, San Diego, 9500 Gilman Dr., La Jolla, CA 92093, USA

**Keywords:** capacitive sensor, sensor array, metamaterials, split-ring-resonator

## Abstract

In this paper, multi-channel capacitive sensor arrays based on microstrip band-stop filters are studied. The sensor arrays can be used to detect the proximity of objects at different positions and directions. Each capacitive sensing structure in the array is connected to an inductive element to form resonance at different frequencies. The resonances are designed to be isolated in the frequency spectrum, such that the change in one channel does not affect resonances at other channels. The inductive element associated with each capacitive sensor can be surface-mounted inductors, integrated microstrip inductors or metamaterial-inspired structures. We show that by using metamaterial split-ring structures coupled to a microstrip line, the quality factor of each resonance can be greatly improved compared to conventional surface-mounted or microstrip meander inductors. With such a microstrip-coupled split-ring design, more sensing elements can be integrated in the same frequency spectrum, and the sensitivity can be greatly improved.

## 1. Introduction

Capacitive sensors, which detect the change in capacitance due to the proximity of a target object, have seen wide use in many industrial applications, including touch sensing, proximity sensing, medical sensing and security scanning [1,2,3,4]. Conventional capacitive sensors are typically excited by an electric signal oscillating at a fixed frequency. A detection circuit monitors the amplitude of the return signal. The signal amplitude increases when there is an object approaching. The responses at different frequencies are often not utilized in conventional capacitive sensors. However, the response of a capacitive sensor is actually frequency dependent. It is worthwhile to explore the frequency response of capacitive sensors to expand the capability of detecting different touching or proximity events. With a capacitive sensor of a single frequency response, only the existence of a touching event can be detected. When the responses at different frequencies are recorded, it is possible to distinguish different touching events. It has been shown that by the sweeping frequency, multiple touching events corresponding to various gestures can indeed be recognized by capacitive sensing [5].

On the other hand, capacitive sensors can be arranged in arrays to extend the application. In most cases, the frequency of the electric signal is fixed, and time-division multiplexing is utilized for the readout [1,2,3]. Frequency multiplexing offers a way to increase the readout efficiency [6,7,8]. It has been shown previously that a target can be detected from the reflected signal of a transmission line with multiple capacitive elements [6,7]. A planar structure based on resonators, in particular metamaterial-based ring resonators, has been proposed for various microwave sensing applications [9,10,11,12,13]. For resonator-based sensors, the quality factor of the resonance is a critical parameter in determining the sensitivity of the sensor. Resonators of a higher quality factor are preferred in most sensing applications. Thus, recent research efforts have been focused on the development of high quality factor sensors [14,15,16]. In particular, when multiple resonators as sensing elements are used in a sensor, the information obtained from different sensing elements would be coupled together in the frequency domain if the quality factors of the resonators are low, resulting in complications in data processing and large uncertainty.

In this paper, we propose capacitive sensor arrays to have multiple elements with decoupled resonances in the frequency domain. Each sensor in the array is embedded in a microstrip line and tuned to distinct resonances. As a simple demonstration, a four-element array with each capacitive patch connected to a surface-mounted inductor is designed and tested. To further improve the performance, meander based on-chip inductors and metamaterial structures can be designed to achieve a higher quality factor of the resonances. Such sensor arrays can be used to detect different proximity/touching events that may occur at different positions.

## 2. Multi-Directional Sensor Array

**Figure 1:**
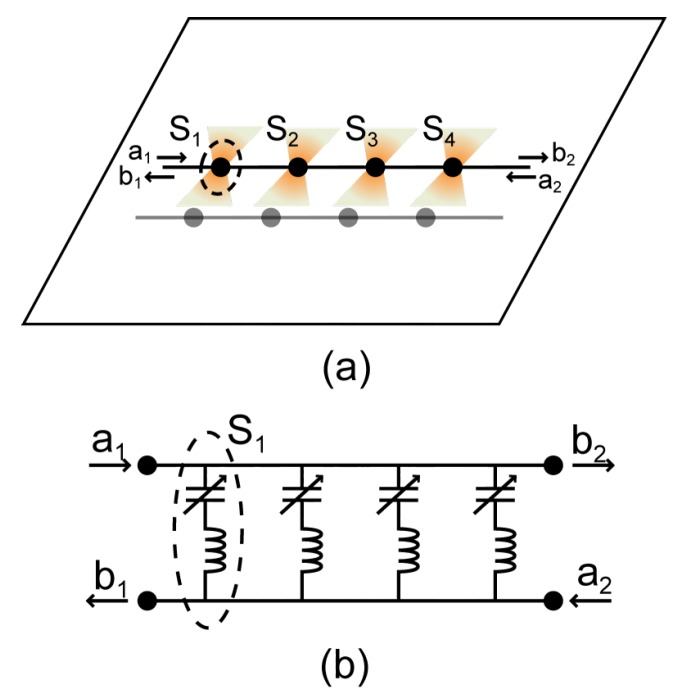
(**a**) An illustration of a capacitive sensor array integrated with a band-stop filter; (**b**) A simplified circuit model for the sensor array. The proximity of the object is detected by the change at the corresponding resonance due to the change in capacitance.

The capacitive sensing method is based on changes in the capacitance value of the sensor due to coupling between the target and the sensor. An inductor-capacitor (LC) resonance can be obtained if the capacitive sensing structure is connected to an inductor. When the inductance value *L* is fixed, the resonant frequency depends solely on the capacitance value of the sensor, as determined by $f_{c}=1/2\pi \sqrt{LC}$. Inductors with different inductance values can be connected to the capacitive sensing element, so that the sensor can operate at different resonant frequencies. Multiple capacitive sensors can be further integrated in a microstrip line and work as an array, with each element tuned to distinct resonant frequencies. This concept is similar to a microwave filter with tunable capacitance values [17,18,19]. As illustrated in Figure 1, when there is a change in capacitance value, the corresponding resonant frequency is shifted accordingly. A change in the transmission coefficient at a particular operating frequency near the resonance is also expected. It is thus possible to detect either the shift in resonant frequency or the change in the transmission coefficient for sensing purposes. The detection in resonant frequency shift can be realized by monitoring the input impedance of the sensor. In this paper, the focus is on the design of sensing structures, and the measurements are performed with a vector network analyzer. However, the functionality of the network analyzer can be realized with sensing circuits to measure the impedance and detect the resonant frequency shift.

The series inductance can be realized in different forms. In the simplest case, the series inductance can be a packaged inductor. On-chip designs, such as meander-shaped inductors, can also be used. Metamaterial structures, such as split-ring resonators, can also be explored. It is desirable to have a resonance with a higher quality factor, such that the neighboring elements can more easily be separated in the frequency spectrum, and more sensing elements can be allocated in a given frequency band. This is seen from the definition of the quality factor $Q=f_{c}/\delta f$, where $f_{c}$ is the resonant frequency and δf is the half-power bandwidth.

**Figure 2:**
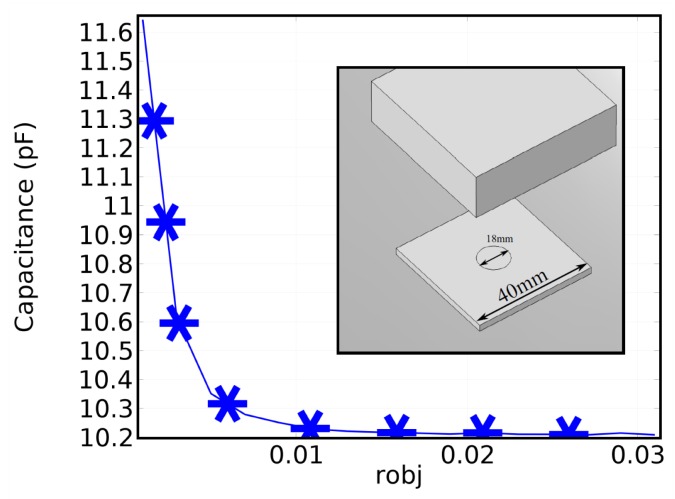
Simulation of the patch sensor and its capacitance change with the distance of an object. The patch has a radius of 9 mm, on FR4 substrate of a size of 40 mm × 40 mm and a thickness of 1.2 mm. Simulations are done in commercial software COMSOL Multiphysics.

We first explore the use of packaged inductors for such a sensor array. Based on the described principle, a four-element proximity sensor array is designed to work in multiple directions. The individual capacitive sensing structure is a circular patch of copper film and a ground plane separated by a dielectric substrate, as shown in Figure 2, The capacitance, as well as the change in capacitance can be calculated when there is an object (a block of wood; Figure 2) approaching the patch. The calculated capacitance values are shown as a function of distance between the target object and the patch. We can see from the figure that the capacitance value increases with the approaching of the object.

**Figure 3:**
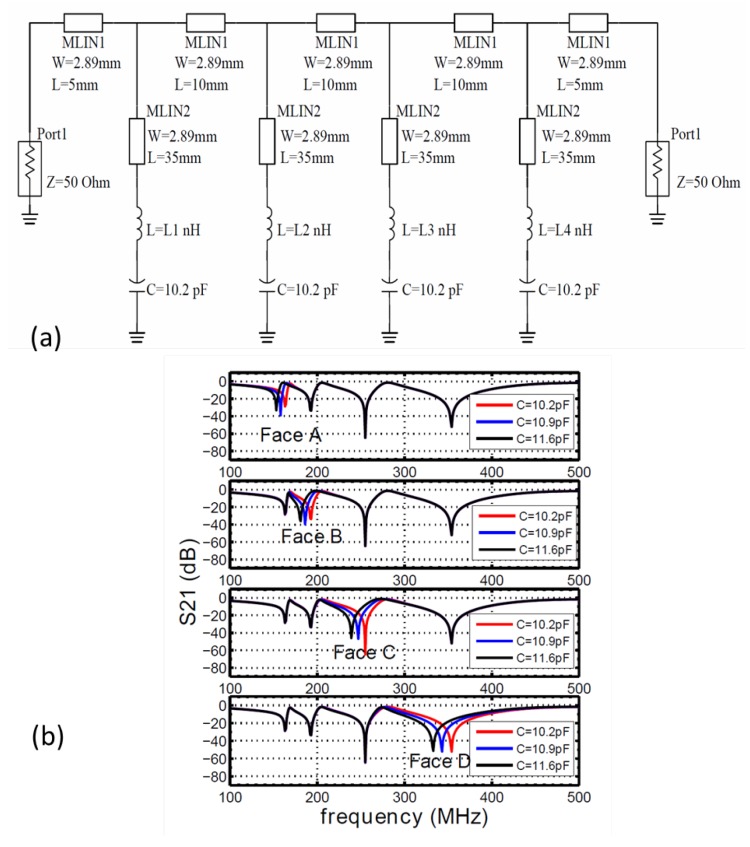
(**a**) The schematic of the circuit simulation. $C_{1}=10.2$ pF; $L_{i}$ (i=1,2,3,4) is 82 nH, 56 nH, 27 nH and 8.2 nH, respectively. (**b**) Transmission spectrum given by circuit simulations. The four sub-figures indicate the change in capacitance at four sensing elements, A, B, C, D, with different inductance values. In each sub-figure, curves with different colors represent different capacitance values.

A circuit model is then built with the simulated capacitance values for a four-element sensor array. The schematic circuit model is shown in Figure 3a. We first fix the capacitance value of 10.2 pF for each capacitor, which is connected to an inductor to form its LC resonance and then connected to the same transmission line. Since the capacitance value is fixed, the resonant frequency is controlled by the corresponding inductor. The inductances for the four elements are chosen in order to have good separation in the frequency domain. The corresponding resonant frequencies are around 174 MHz, 211 MHz, 303 MHz and 550 MHz, respectively. All sensing structures are of the same dimension as simulated in COMSOL (Figure 2). In the circuit simulation, all of the capacitors and inductors are assumed to be ideal components for simplicity. Once the circuit model is built, we vary the value of each capacitor from 10.2 pF to 11.6 pF in the simulation, to mimic the approaching of the wood block, as shown in Figure 2. The results of circuit simulations are shown in Figure 3b. For each sub-figure, one capacitor is tuned while keeping all others fixed, and the transmission coefficient is plotted with the values of the tuned capacitor. From the figures, we can see that the change of one capacitor only affects the transmission at the corresponding stop band and does not disturb other bands. It should also be noted that the resonant frequencies shown in the circuit simulation deviate from the previous calculation based on ideal capacitance *C* and inductance *L* values. This is mainly due to the transmission line effect that is neglected in the simple LC calculation. However, the isolation in resonant frequencies is not affected by the shifting in resonance, and the independent control of resonant frequency is still valid.

**Figure 4:**
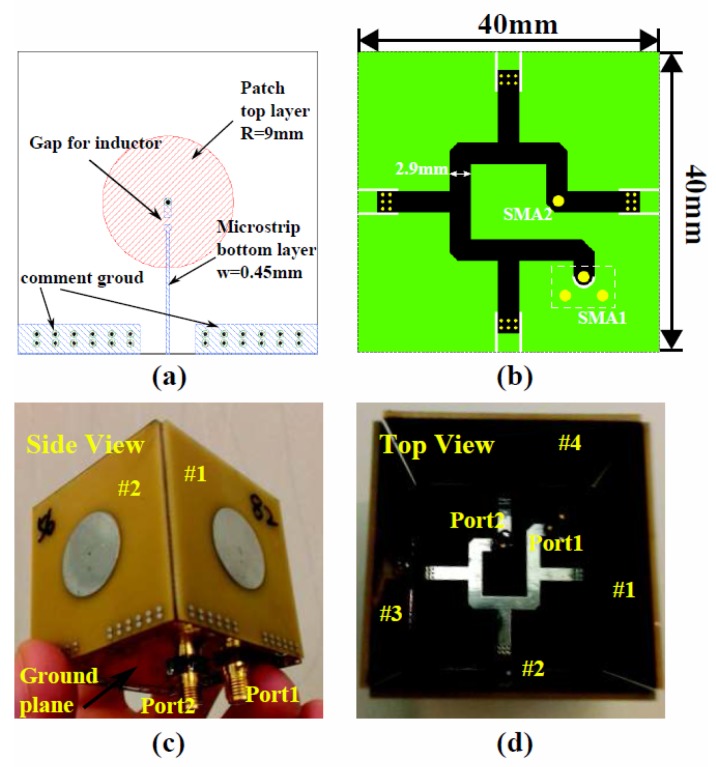
The designed layout and the fabricated sensor. (**a**) The layout of the sensor element; (**b**) the layout of the feeding network, with the top layer shown in black and the bottom layer shown in green; (**c**,**d**) pictures of the fabricated sensor.

In the next step, the sensor elements and feeding networks are designed using simulation software ADS, as shown in Figure 4a,b. The sensing elements are designed on a three-layer printed circuit board (PCB), with FR-4as the substrate. The top layer of the sensing patches is connected to a 50 Ω transmission line located on the bottom layer of the PCB with a via, while the ground plane sits in the middle layer, with a 1.2-mm spacing between the top layer and a 0.3-mm spacing between the bottom layer.

A feeding network is also designed for the four sensing elements. A folded design is utilized, since the sensing elements are assembled in various directions. The geometrical parameters of the design are provided in Figure 4a. After fabricating the circuit boards, four capacitive sensing elements are assembled, so that they form a cube. The transmission line of the feeding network is connected to each of the four sensing elements from inside the cube, and the ground planes of the four elements are soldered together. Pictures of the assembled capacitive sensor array are shown in Figure 4c,d.

**Figure 5:**
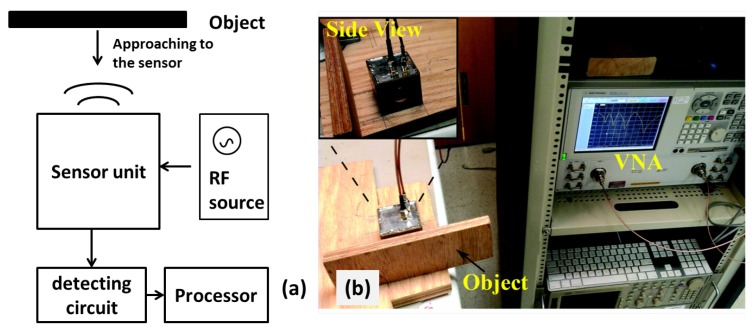
(**a**) Measurement block diagram; (**b**) experiment setup with the vector network analyzer.

**Figure 6:**
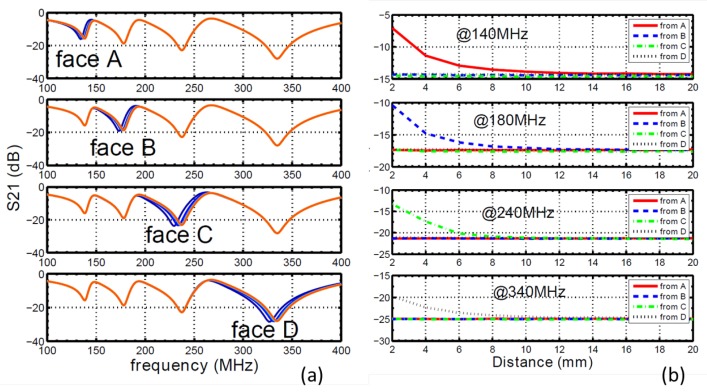
(**a**) Measured transmission spectrum. The four sub-figures indicate the change in capacitance at four sensing elements with different inductance values. In each sub-figure, curves with different colors represent different distances between the target and sensing element; (**b**) The four sub-figures are for the $S_{21}$ at different frequencies. The four curves in each sub-figure represent the object coming from four different directions.

Figure 5a shows the block diagram to realize the sensing functionality. The sensor is excited by an RF source, and the return signal from the sensor is sent to a detecting circuit, which is then processed by a processor. The proximity of an object is detected by measuring the difference in the return signal. The focus of this paper is on the sensing structure design, and the signal generation and detection are all realized by a vector network analyzer, as shown in Figure 5b. A target object (block of wood) is placed to face one of the sensors. The distance between the target object and the sensor is changed from 2 mm to 20 m with a step size of 2 mm, and the S-parameters for each case are measured with the network analyzer. Then, the target object is moved to a different direction and faces another sensor, and the same process of measurements is repeated for each case. In Figure 6a, we show the measured magnitude of $S_{21}$ of different cases, with each sub-figure indicating an object coming from a different direction and each curve representing measurements at different distances between the object and sensor. By comparing the four sub-figures, we can see that the approaching of an object in one direction only causes the resonant frequency to shift to the corresponding sensor, while not affecting all other resonances.

If we compare the measurement results with the simulation results, as shown in Figure 3b, we can see that the two figures agree very well with each other. There are small discrepancies in the resonant frequency, as well as the sharpness of the curve for all cases. This is because in the circuit simulations, ideal capacitors and inductors are assumed. In reality, the components are not ideal: there is small parasitic inductance associated with each capacitive patch; there is parasitic capacitance and resistance associated with each on-chip inductor; there are additional parasitics introduced during the soldering process. On the other hand, the quality factor of resonances observed in the measurement results (Figure 6a) is significantly lower than seen in the simulation results. This is again due to the simplified model in circuit simulations, neglecting ohmic losses induced in each element. Dielectric loss from the circuit board substrate and ohmic loss from metallic structures all affect the quality factor of a resonance.

Other than tracking the change in resonant frequency, we can also detect the object by tracking the magnitude of $S_{21}$ at a frequency close to the resonance. As shown in Figure 6b, we choose one frequency around each of the four resonances for $S_{21}$ magnitude analysis. The change of $S_{21}$ magnitude at these frequencies *versus* the distance between the target object and sensor is plotted for cases when the object is approaching from four different directions. At each particular frequency, only one curve shows an observable change in $S_{21}$ at different distances, while all others remain the same. This again indicates that each frequency is only sensitive to objects in the corresponding direction. The sensing range can also be estimated from Figure 6b. The uncertainty in transmission magnitude measurement can be controlled to be under 0.1 dB according to the manufacturer of the network analyzer, Agilent (now KeySight [20]). Thus, it is reasonable to assume the uncertainty to be 0.5 dB to estimate the sensing range. According to the measurement data, the sensing range of our sensor is around 10 mm. Furthermore, within the sensing range, a larger than 0.5 dB/mm change in magnitude is obtained, indicating that sub-millimeter resolution can be achieved with the sensor.

**Figure 7:**
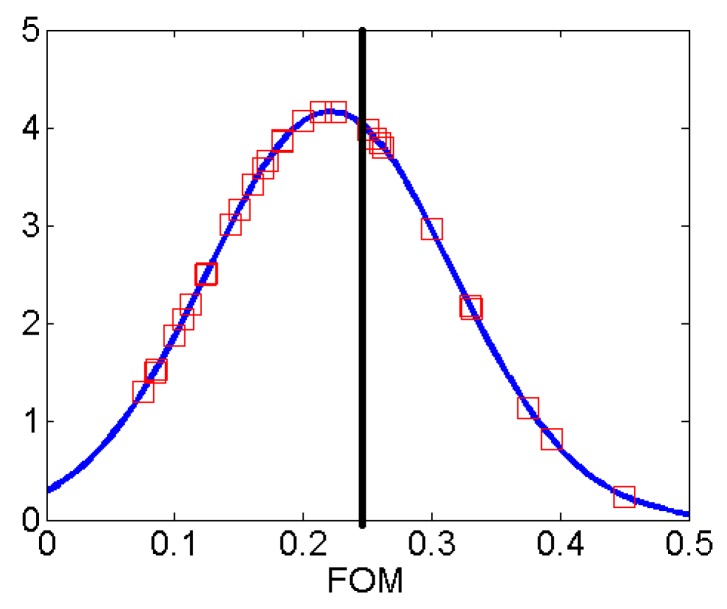
The comparison of the sensor figure-of-merit (FOM) with commercial sensors. All data points are fitted in a Gaussian distribution, with mean 0.221 and a standard deviation of 0.096. Squares are FOM from commercial sensors, and the black line indicates the FOM of the designed sensor.

The sensing range is compared to state-of-the-art proximity sensors from major sensor manufacturers and vendors, including OMRON, IFM, SICK and AutomationDirect. To have a fair comparison, the nominal sensing range $S_{n}$ is adjusted with a correction factor, which is determined by the material type of the target object. For a piece of wood, the correction factor is taken as 0.45. Thus, the corrected sensing range is $S_{corrected}=0.45S_{n}$. Furthermore, in general, the sensing range is proportional to the physical size of the sensing area. Thus, we define the figure-of-merit (FOM) for sensing performance using the ratio of the corrected sensing range and the effective dimension of the sensor $D_{eff}$, which is defined as the geometric mean of the two dimensions of the sensor area. $FOM=S_{corrected}/D_{eff}$. For our sensor, FOM=0.45×10/18=0.25. From our survey, the FOM obtained from commercially available sensors ranges from 0.1 to 0.4, as shown in [21]. If we plot all of the FOMs of commercial sensors and our design, we can get a direct comparison, as shown in Figure 7. All of the FOM data points are fitted in a Gaussian curve, with a mean of 0.221 and a standard deviation of 0.096. The proposed sensor has a FOM larger than most of the available sensors, with the added feature of utilizing the resonances in the frequency domain, which is not available in those commercial sensors.

## 3. Linear Sensor Array

The same design principle can be applied for various configurations in a sensing array. For instance, linear arrays based on microstrip structures can be designed, which can be useful for linear position sensing. To demonstrate the idea, a linear array of four elements is designed.

**Figure 8:**
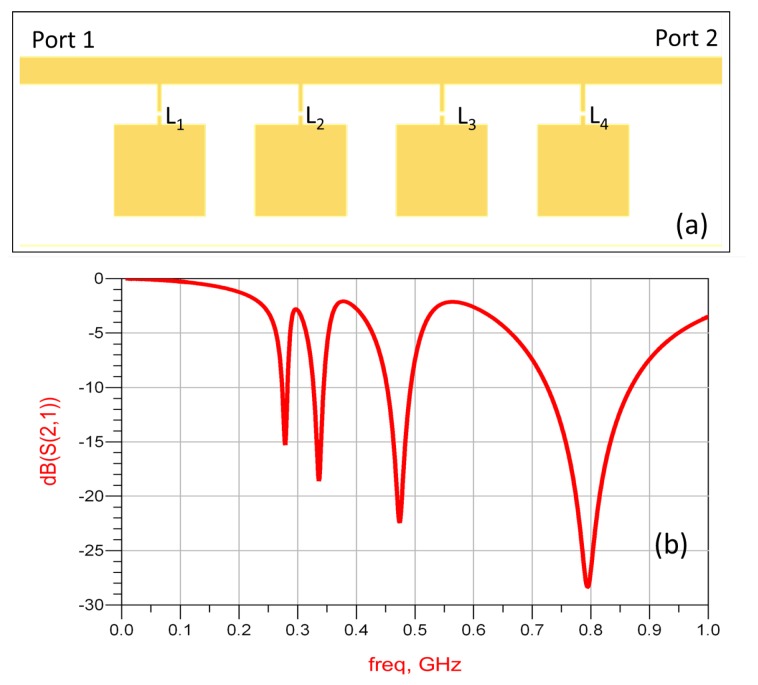
(**a**) The simulated microstrip structure in ADS; (**b**) simulated transmission spectrum.

As shown in Figure 8, each element has a 10 mm by 10 mm square patch as the sensing structure, integrated in a two-port microstrip line, through a series inductance; the values are $L_{1}=100$ nH. $L_{2}=68$ nH, $L_{3}=33$ nH, and $L_{4}=10$ nH, respectively. The model is simulated in ADS, and the transmission spectrum is shown in Figure 8b. Sharp resonances are observed for each element.

**Figure 9:**
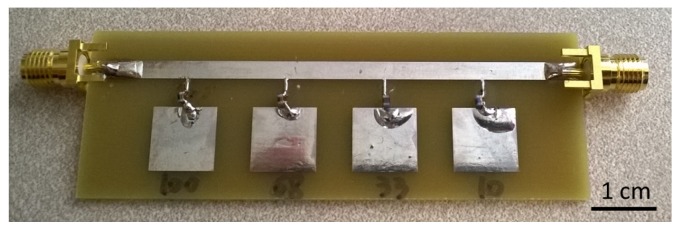
Fabricated capacitive sensor array on a microstrip line with connected surface mount inductors.

**Figure 10:**
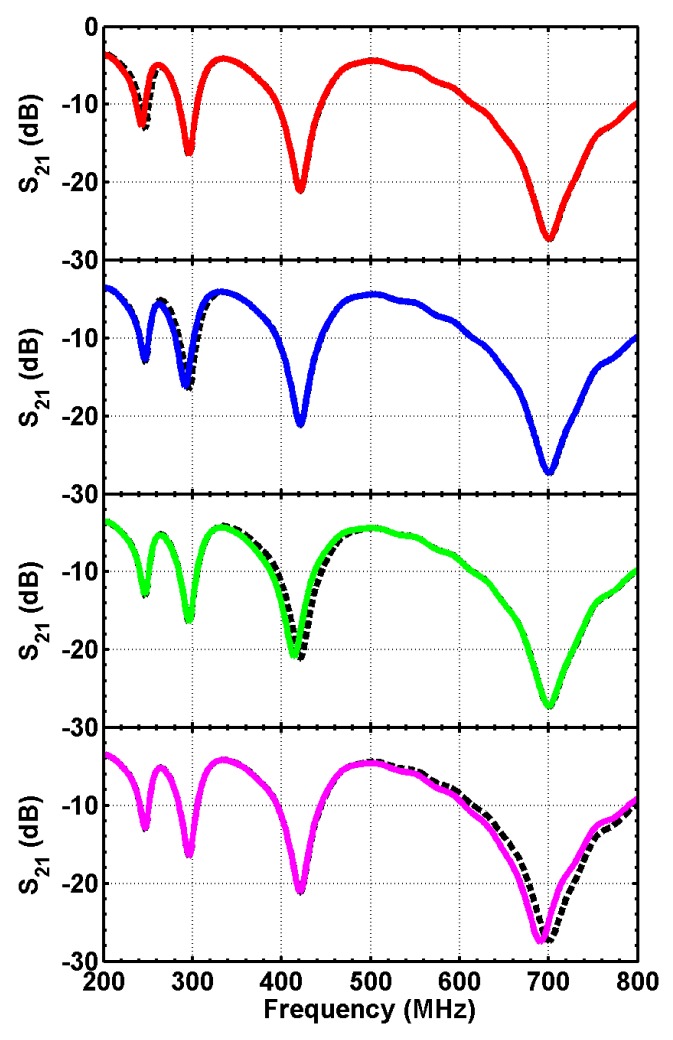
Measured transmission spectrum when the target is facing each of the four sensors. The dashed line is the measurement from the unloaded sensors.

**Figure 11:**
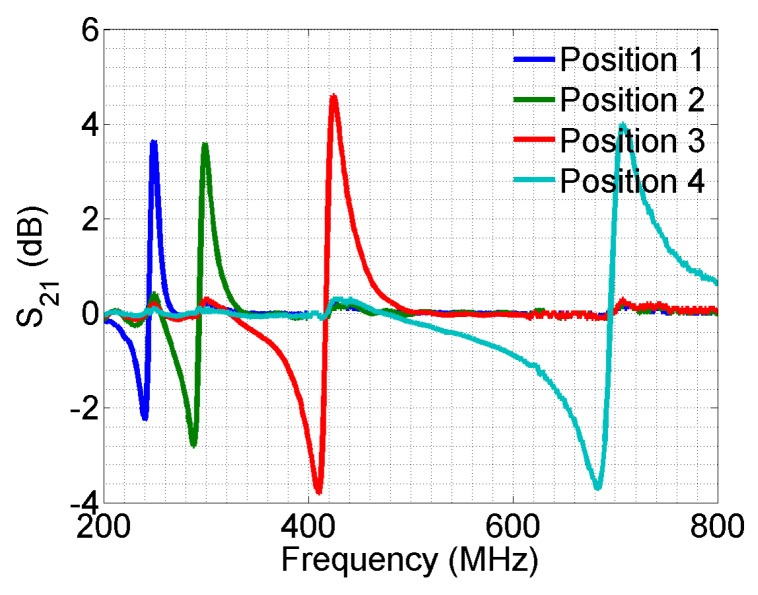
The transmission spectrum with the target at the four different positions, normalized by the unloaded case.

The design is then fabricated on a printed circuit board, with substrate FR4 of thickness 1.6 mm (Figure 9). Surface-mounted inductors are used with values the same as used in ADS simulations. The transmission spectrum of the sensor array is measured with a vector network analyzer. The unloaded sensor is first measured, and the transmission spectrum is used as a baseline. A piece of wood block (dielectric constant of two, loss tangent of 0.02) of 10 mm wide is used as the target object. The transmission spectrum is measured when the target object is facing each of the sensor patches and at a distance of 5 mm away from the surface. The measurement results are plotted in Figure 10. The measurement results agree very well with the ADS simulation (Figure 8). Furthermore, when the target object is at a particular position, only the corresponding resonance is disturbed, with all other parts of the spectrum unchanged. Such a change in the transmission spectrum is more clearly observed when plotting the difference between each measurement and the baseline. As seen from Figure 11, at least a 3.5-dB change is observed at the corresponding resonance for all four measurements. Away from the resonance, the transmission is basically the same as the baseline case without the presence of a target object. The study shows that the sensing elements integrated in a single microstrip line can be well isolated in the frequency spectrum and used as independent channels.

**Figure 12:**
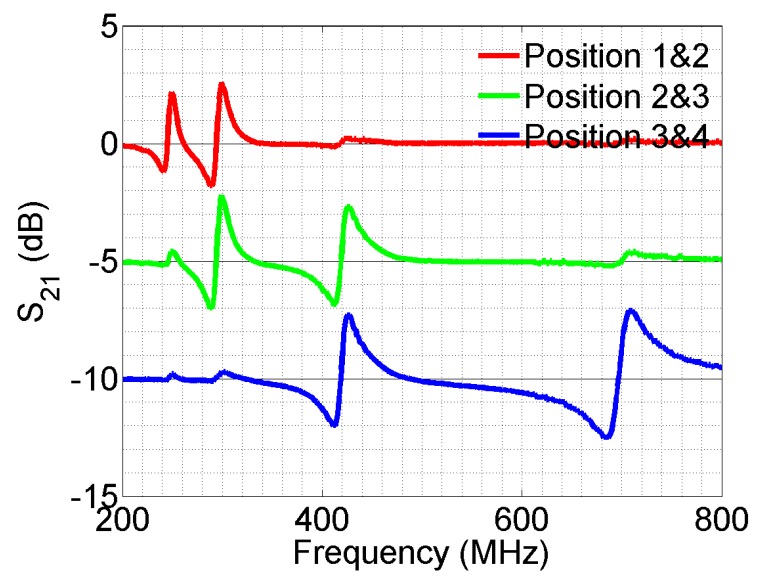
The transmission spectrum with the target at two positions at the same time, normalized by the unloaded case. Additional curves are moved down 5 dB on the y-axis each time to separate the overlapping region for better illustration.

To further demonstrate the sensing principle, another set of measurements is performed, with target objects approaching two sensors at the same time. We measured the case when Sensor Numbers 1 and 2, 2 and 3 and 3 and 4 are loaded, respectively. In all measurements, the distance between the target and sensor is the same for comparison. The results are shown in Figure 12. Since the response is almost identical when a sensor is loaded in different cases, the curves are separated on the *y*-axis by 5 dB each for better illustration. Again, only the resonances associated with loaded sensors are changed significantly. It is shown that the array can be used to detect multiple objects at the same time. With more elements in the sensor array, various proximity/touch events can be detected.

The use of inductive components brings resonance and adds freedom in sensor design in the frequency domain. As mentioned before, different ways of realizing inductive components can be used. Instead of surface-mounted inductors, on-chip designs, such as spirals and meanders, can also be adopted, such that all components in the sensors can be drawn on the same printed circuit board without the need for soldering, which eases the fabrication process. By changing the geometrical parameters of the on-chip designs, the inductance values can be easily controlled. Alternatively, the inductive components can be coupled to the microstrip line, instead of directly connected to it.

## 4. Split-Ring Coupled Sensor Array

Recently, metamaterial structures have been used in many microwave applications [22]. Metamaterial-based resonators can have a compact size and a high quality factor; when coupled to microstrip lines, new microwave filters can be built. They have also been considered for sensing applications. In [23,24,25], a multi-resonance sensing method has been demonstrated using structures based on split-ring resonators (SRRs). With the help of SRRs, the localized electric field is greatly enhanced in the very small gap region within the SRR, such that the sensitivity is largely increased. However, the sensing area is limited due to the sensing principle, which relies on the subtle change in the dielectric constant of the small gap region. Plus, the object has to be in contact with the sensor. Here, we show that by combining split-rings with capacitive sensing elements, sharp resonances can be designed, such that more channels can be potentially included in the same bandwidth.

**Figure 13:**
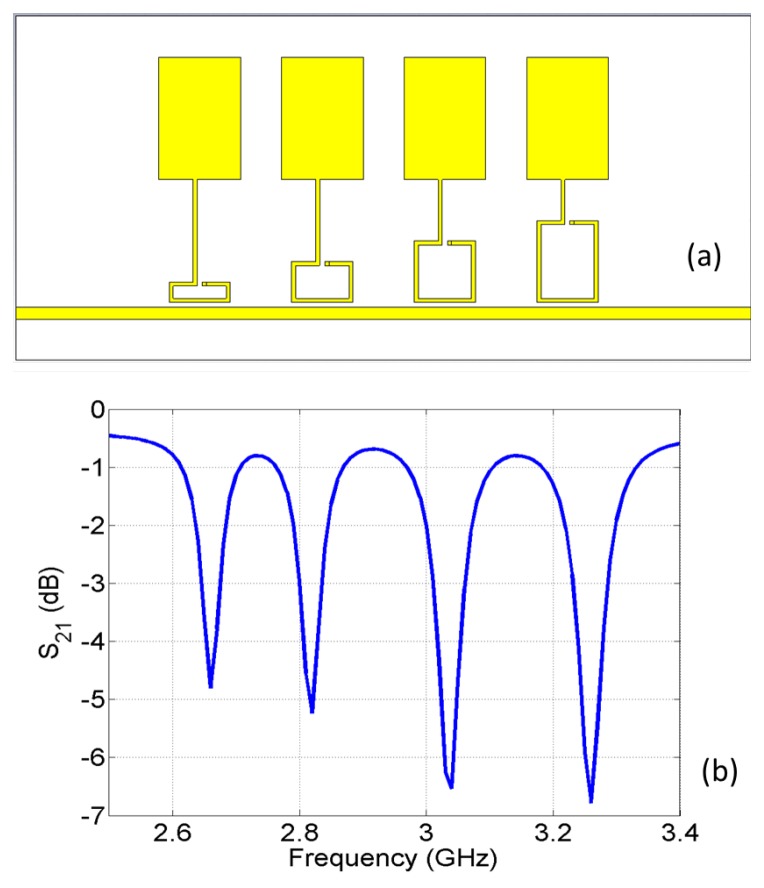
(**a**) A model of a capacitive sensor array coupled to a microstrip line through split rings of different dimensions; (**b**) simulated transmission spectrum of the unloaded line.

**Figure 14:**
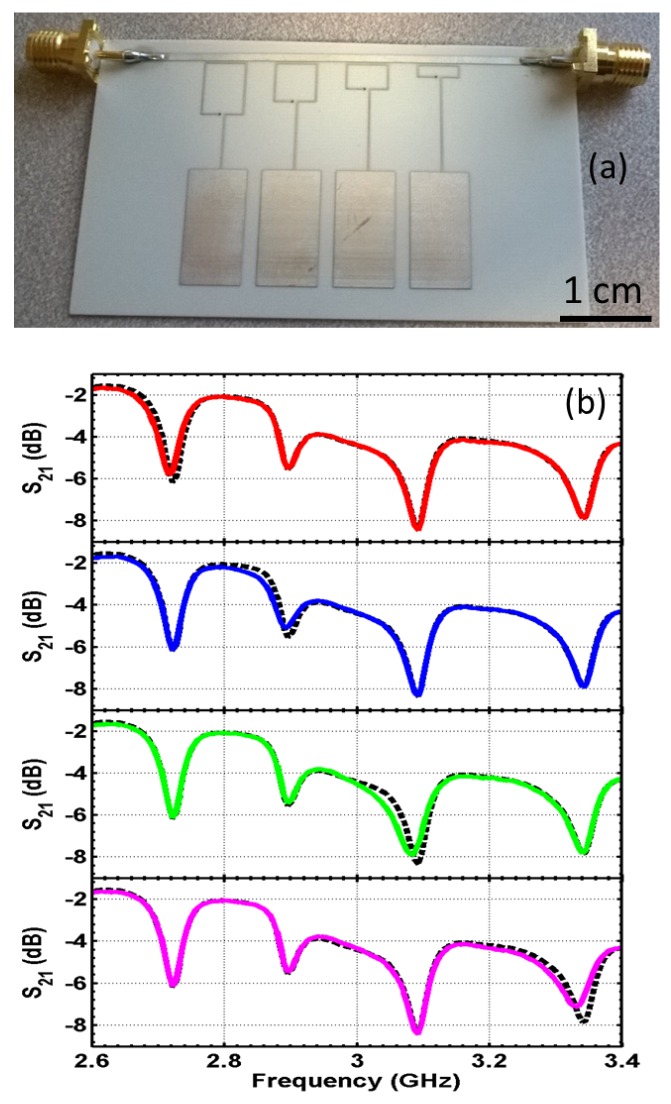
(**a**) The fabricated capacitive sensor array with microstrip-coupled split-rings; (**b**) experiment result on the fabricated sensor array. The four subfigures show the transmission spectrum with the object present at each of the capacitive sensors (colored solid curve), compared to transmission without the object (black dashed curve).

A four-element sensor array is designed with capacitive patches connected to single-turn split-rings and coupled to a microstrip line, as shown in Figure 13a. The transmission coefficient of the line is simulated and plotted in Figure 13b. Four distinct resonances are seen in the spectrum and show the band stop feature. The design is then fabricated (Figure 14a) and measured experimentally (Figure 14b). Again, the transmission spectrum with the object present at each of the capacitive sensors is measured and compared to the transmission without the object (baseline case). Similar to the result shown in Figure 10, the transmission coefficient changes around the resonant frequency of the corresponding sensor when the target object is approaching the surface of the sensor. Other frequencies in the band are not perturbed. Further experiments and design optimizations will help further improve the performance of the sensor array.

Compared to on-chip inductor-based designs, the SRR coupled sensor has more design flexibility in tuning the resonance frequency of the sensor and achieves a much higher quality factor. Again, the quality factor can be obtained from the ratio of the resonant frequency $f_{c}$ and the half-power bandwidth of the resonance δf. The quality factors of the fabricated sensors with inductors mounted range from five to 12; while the SRR coupled sensor can achieve a quality factor of around 90. This shows that a higher quality factor can indeed be achieved with the metamaterial approach. Furthermore, the SRR-based design agrees with most of the metamaterial-based microwave sensor designs toward a higher quality factor, with typical quality factors varying between 50 and 100 [9,10,11,12,13]. The resonances from the SRR coupled sensor array are within the band between 2.6 and 3.3 GHz, with a fractional bandwidth of about 12%, while for the design with surface-mounted inductors, the fractional bandwidth is over 100% (frequency band between 0.25 and 0.85 GHz). The higher the quality factor, the more sensors we can fit in for a given frequency band.

Note that the several designs presented in this paper are mainly for proof-of-concept purposes. There are no specific criteria for these designs, including the operating frequency. The first design is to demonstrate the multi-directional sensing capability with one array of capacitive sensing elements; the second design is to demonstrate the sensing capability while the capacitive sensors are assembled in a linear array; the last design provides an alternative to on-chip inductors in the SRR coupled approach for LC resonance of a higher quality factor. In order to achieve specific sensing purposes, the principles introduced in this paper can be applied for sensor structures more suitable for the required operating conditions.

## 5. Conclusions

In summary, we studied multi-channel capacitive sensor arrays based on multiple resonances. Each capacitive sensing structure in the array is connected to an inductive element to form a resonance at different frequencies. The resonances are designed to be isolated in the frequency band, such that the change in one channel does not disturb resonances at other channels. The inductive element associated with each capacitive sensor can be surface-mounted inductors, integrated microstrip inductors or metamaterial-based structures. We show that by using split-ring structures coupled to a microstrip line, the quality factor of each resonance can be greatly improved compared to conventional designs. With such a microstrip-coupled split-ring design, more sensing elements can be integrated in the same bandwidth, which helps improve the capability and performance of such sensor arrays.
